# Has stakeholder participation in health facility governing committees promoted social accountability? A qualitative study in Tanzania

**DOI:** 10.1080/16549716.2024.2432067

**Published:** 2024-11-25

**Authors:** Hussein Athuman Kapuya, Stephen Oswald Maluka, Anna-Karin Hurtig, Miguel San Sebastian

**Affiliations:** a Dar es Salaam University College of Education, University of Dar es Salaam, Dar es Salaam, Tanzania; b Department of Epidemiology and Global Health, Umeå University, Umeå, Sweden

**Keywords:** Stakeholder participation, social accountability, health facility governing committees, health system, Tanzania

## Abstract

**Background:**

Since the 1990s, Tanzania has actively encouraged stakeholder participation in health services through Health Facility Governing Committees (HFGCs) to promote social accountability within its broader health system reforms. While previous studies have explored the functionality of the HFGCs, this study aimed to understand whether stakeholder participation in the HFGCs contributes to promoting social accountability in the health system.

**Methods:**

Between July and October 2022, a qualitative study in two districts of Tanzania was carried out. Selected participants, knowledgeable about HFGCs, included facility managers, HFGC members representing diverse stakeholders in the committees, local government leaders, district health secretaries, and religious leaders. Twenty in-depth interviews were conducted and analysed thematically.

**Results:**

Findings revealed low stakeholder participation in the committees´ activities, partly due to the committees´ inability to effectively sensitize and mobilize them. Inadequate support from local government leaders and the dominance of the facility managers in the committees´ activities, also affected the committees´ role as promoters of social accountability.

**Conclusion:**

The HFGCs in Tanzania have faced challenges in promoting social accountability due to weak stakeholder participation. Key issues include limited awareness of HFGCs, inadequate mobilization, and insufficient training, supervision, and guidelines from district councils. Additionally, flawed election processes, leadership interference, lack of political support, and limited financial resources undermined the committees’ effectiveness. To improve engagement, district councils should enhance stakeholder sensitization, build HFGC capacity, ensure that facilities allocate 5% of their budgets for committee activities, monitor performance, and encourage local government support for HFGCs.

## Paper context

- **Main findings:** The contribution of stakeholders in Health Facility Governing Committees (HFGCs) to promote social accountability in Tanzania´s local system was very weak. Stakeholders´ awareness about their roles and participation in the committees´ activities was too low to promote social accountability. There was a lack of political support from local government, facility heads overextended in their roles, and supportive groups like Council Health Management Teams (CHMTs), Council Health Services Board (CHSBs), NGOs, and FBOs failed to effectively assist the HFGCs in their roles.
- **Added knowledge:** Although there is some understanding on how HFGCs operate, this paper fills a knowledge gap regarding the contribution of various key stakeholders in the committees in promoting social accountability.
- **Global health impact for policy action:** Policy and intervention efforts that promote stakeholder participation in HFGCs are required to significantly promote social accountability in the local health systems.

## Background

Stakeholders encompass any group or individual capable of influencing or being affected by an organizational goal [1]. Within the governance frameworks of public agencies, stakeholders’ engagement has emerged as a pivotal element [2]. It serves as a cornerstone of social accountability, empowering them to shape and share control over priority setting, policy formation, resource allocation, and access to public services [3]. Through stakeholders’ participation, social accountability endeavours to fortify governance from the ground up, fostering inclusive decision-making that incorporates a spectrum of perspectives, interests, and needs [4]. This inclusive approach fosters stakeholder buy-in, enhances trust, and deepens comprehension of the diverse interests, values, and requirements of the involved parties [5]. Globally, numerous countries have embarked on concerted initiatives to establish mechanisms for stakeholders’ involvement, such as client stakeholders’ bodies and user panels, to advance social accountability within the sphere of health-sector governance [2]. Stakeholder participation in health through stakeholders´ bodies originates from the Alma Ata Declaration in 1978 [6]. The declaration advocated for primary health care through maximum community participation to address health inequalities and ensure state responsibility for citizens [6].

Many low- and middle-income countries (LMICs) have recognized that enhancing stakeholder involvement in health care is a strategic avenue with potential benefits, including advancing democratization processes, realizing cost efficiencies, and optimizing the utilization of existing human and financial resources [7]. Particularly in Africa, the movement towards fostering social accountability mechanisms for health care participation dates back to the Bamako initiative of 1987 [8]. This initiative advocated for the establishment of health committees by states to promote community engagement, giving the community a voice in accessing health-care services, influencing planning activities, and managing funds [8,9]. In response, several African countries have taken this further by forming health committees with representation from diverse interest groups [10]. While the implementation of some of these initiatives has been successful, others have encountered significant challenges [11]. For instance, Malawi has identified the lack of stakeholder-led initiatives and disparities in decision-making power among health-care stakeholders as significant governance obstacles hindering public participation in the health-care system [12]. In South Africa, issues such as lack of formal recognition and unreliable allowances have impacted representation within committees, leading to some members becoming tired of volunteering [13]. Gender exclusion has also emerged as another notable concern, particularly affecting the participation of historically marginalized groups [13].

### Social accountability initiatives in the local health system in Tanzania

Tanzania has actively pursued initiatives to enhance social accountability within its local health system since the early 1990s, prioritizing the establishment of health committees as pivotal bodies for community engagement. A significant step was the enforcement of the National Health Policy in 1990, which mandated every village to appoint a minimum of two community health workers (CHWs) to promote community participation in the implementation of primary health care (PHC) [14]. These CHWs played a crucial role in bridging the gap between the community and the health facilities. Moreover, the policy emphasized the importance of collaboration with various stakeholders, including community groups, religious bodies, charitable organizations, and the private sector, underscoring a holistic approach [14]. To enhance stakeholder participation in managing PHC services, the government established the Health Facility Governing Committees (HFGCs) in 1999 [15]. These committees’ mandate was to oversee PHC service provision according to the guidelines set forth in 2001. This guideline’s review in 2013 had as one of its objectives to direct the structure of health services’ management bodies to promote transparency and accountability in the management and use of public resources [16]. The guidelines mandated the inclusion of representatives of communities, facility staff, private health service providers, faith-based organizations (FBOs), or non-governmental organizations (NGOs), and local government authorities [16] as members of the HFGCs. A chairperson, selected from community representatives, should head the committee, while the manager of the facility assumes the role of the secretary by default. To ensure gender diversity, at least one of the three community members should be a woman [16].

### The Tanzanian health system

Since the 1990s, Tanzania has implemented a decentralized health system structured into three tiers: local, regional, and national [14,17]. This system follows a referral pyramid model, starting with dispensaries at the village level, extending up to the ward with health centres, and then extending to the district level with district hospitals (25). Furthermore, the system progresses to the regional level with regional referral hospitals, and culminates at the national level with national and specialized hospitals [17]. The primary objective behind decentralization is to foster the engagement of diverse stakeholders in overseeing health-care services across different tiers of the system [14,17,18]. To enhance stakeholder involvement in HFGCs and thereby promote social accountability within the local health system, various stakeholders had distinct roles assigned to them, as outlined in Table 1.

**Table 1.** Stakeholders’ roles in the HFGCs.

Stakeholder	Role
Facility users (community)	● To participate in selecting HFGC representatives through the general assembly. ● To take an active role in identifying challenges, prioritizing needs, planning initiatives, monitoring operations, evaluating service provision, and receiving updates from their HFGC representatives. ● To play a pivotal role in implementing the Annual Collaborative Facility Plan aimed at enhancing health-care services across dispensaries, health centres, and district hospitals.
The local government authorities (*mtaa*/village, ward and the (district, municipal, town and city) council levels.	● At the mtaa/village government authority level, leaders are required: ● To supervise the formulation of the HFGCs. ● To appoint a representative to the HFGC who will serve as the liaison between the *mtaa*/village government and the committee. ● To ensure active participation of the community and stakeholders in providing input and ideas for improving and operating health services at the local facility, facilitated by its village council health services committee (VCHSC). ●To foster and enhance relationships among various health-related committees within their jurisdiction through the VCHSC and the members representing the village/*mtaa* government to the HFGC. ● To ensure the community is involved in implementing the Annual Collaborative Dispensary Plan for health service provision, facilitated by the HFGCs through its VCHSC. ● To ensure the HFGCs work with the communities and other stakeholders to ensure the local facilities have sufficient infrastructures such as staff houses, treatment rooms, water, and other necessities. ● To ensure the entire community participates in improving the management and delivery of health services at their dispensary.
	● At the ward local government authority level, it is a request that leaders: ● To supervise the formulation of the HFGCs. ● To appoint a member to represent the ward government to the HFGC. ● To foster a supportive relationship between the ward government and the committees, facilitating effective collaboration and communication through the HFGC members representing the ward government. ● To ensure active community and other stakeholders’ participation in HFGCs both at their dispensaries and health centres.
	● At (district, municipal, town, and city) council, leaders are required: ● To ensure the formation of the HFGCs within the councils. ● To ensure that HFGCs within the council collaborate with the community and other stakeholders in managing and taking ownership of health facilities. ● To ensure that HFGCs have a conducive working environment and to promote social accountability. ● To supervise the facilities and the HFGCs in the council through the Council Health Services Board (CHSB). ● To offer technical advice to all HFGCs in the council through the CHMT. ● Each council is required to have a participatory annual health plan that is obtained by combining all the facility plans within the district ● Each district council through its CHSB is required to support the committees, especially in staff supervision matters [16].
Non-Governmental Organizations (NGOs)/ Faith-based Organizations (FBOs)	● To participate in electing their HFGC representatives through special meetings organized by the local government leaders on behalf of the District Directors. ● To provide resources to the HFGCs to facilitate the development of health-care facilities. The HFGC members representing the NGOs/FBOs are required to cultivate a working relationship between the HFGCs and NGOs/FBOs primarily for financial support.
Private health service providers	● To participate in electing their HFGC members through special meetings organized by the local government leaders on behalf of the District Directors. ● To assist the HFGCs in delivering health services. HFGC members representing the private health service providers are tasked with a duty to foster and enhance such a working relationship.
Health Facility Management Teams (HFMTs)	● To act upon the resolutions passed by the HFGCs. The person in charge of the facilities is responsible for linking the HFGCs and HFMTs. ● To provide feedback on the approved annual facility plan to HFGCs.

Sources: [17,19].

### The role of HFGCs in promoting social accountability in Tanzania

The HFGCs have a complex structure and demanding responsibilities. These committees’ tasks include approving facility plans and budgets, sourcing resources from diverse channels, and receiving quarterly and annual implementation updates from the Health Facility Management Teams (HFMTs) [16,20]. The HFMT in each facility is comprised of heads of sections chaired by the facility manager. Other committee tasks include addressing community concerns and promoting participation in the community health fund (CHF), a voluntary community-based health insurance initiative [16,20]. To fulfil these obligations, a variety of stakeholders represented within the HFGCs should provide support to the committees in their activities as described in Table 1. The members of the HFGCs were required to promote relationships between the committees they served and the stakeholder groups they represented [16]. Additionally, some key supporting stakeholders from the district councils were tasked with various roles to assist the committees as outlined in Table 1.

Previous studies examining the functioning of the HFGCs have highlighted their inadequate performance in advancing social accountability [20–22]. The major reason provided for this failure includes the limited awareness among members of the committees with regard to their roles [21–23]. Additional difficulties arose from insufficient financial incentives for the committees, lack of supervision from the district health managers, and inadequate training [21].

Although there is some understanding of how HFGCs operate, there remains a gap in knowledge regarding the engagement of various stakeholder groups in their functions. Thus, this study aimed to explore whether stakeholder participation in the HFGCs was really able to promote social accountability in two districts of Tanzania [24].

## Conceptual framework

The study applied the social accountability framework by Brinkerhoff and Wetterberg [24], to guide the data collection and to structure the discussion of the findings. Social accountability refers to a participatory process that engages citizens to hold public officials accountable for service delivery [11,25]. In this study, ‘citizens’ refers to stakeholders who are expected to engage with the committees, while ‘public officials’ refers to the Ministry of Health represented by the health facility staff. The framework suggests three key conditions for promoting social accountability [24]. The first condition focuses on how to enable the community receiving poor services to mobilize, come together, and demand improvements. The second condition examines the extent to which the authority providing services has the ability to provide them, where authority and resources are highlighted. The third condition explores the contextual factors, asking what is the overall context of authority–society relations, and to what extent does the community trust and legitimize the authority in providing such services?

## Methods

### Study setting

This study was carried out in the Handeni and Mbarali districts, located in the north-eastern and south-western parts of Tanzania, respectively. We selected these two districts based on their performance in the 2018 Star Rating [19], whereby Mbarali received the rating of a high-performing district and Handeni a low-performing one. The Star Rating Scale was a nationwide survey conducted by the Ministry of Health assessing how socially accountable the facilities (dispensaries, health centres, and hospitals) were [19]. The Star Rating Scale assessed, among other things, whether facilities had functioning health committees and whether health workers involved the community in managing the facilities [19]. Handeni, one of the 11 districts of the Tanga region, has a total population of 276,646 [26]. The major economic activities in the district include livestock farming, hunting and fishing, forestry resources, and subsistence farming [26]. The district has one hospital, one health centre, and five dispensaries. Mbarali is one of the seven districts of the Mbeya region with an approximate population of 300,517 people in 2012 [26]. The main economic activities in Mbarali include agriculture, fishing, forestry, mining, and business, and it is a famous area for rice farming. The district has one hospital, five public health centres, and 34 dispensaries.

### Study design and participants

We carried out this qualitative study between July and October 2022. The study was part of a larger project related to social accountability at the local level of the health system in Tanzania implemented by the Dar es Salaam University College of Education (DUCE) in collaboration with Umeå University, Sweden, and the President’s Office Regional Administration and Local Government (PO-RALG) in Tanzania.

We purposively selected participants from diverse stakeholders based on their knowledge and involvement in the HFGCs. From the two districts, participants included the facility manager, the HFGC chairpersons and members representing diverse stakeholder groups such as the drug vendors/pharmacists, facility users, FBOs/NGOs, and private health service providers. Others included local government leaders, district health secretaries, and religious leaders. All the targeted participants willingly accepted to participate in the interviews. Table 2 shows the participants from the two districts.

**Table 2.** Study participants by district.

Study participants	Handeni	Mbarali
Facility managers (secretaries)	2	2
Chairpersons of HFGCs	2	2
Members of HFGCs	2	3
Local government leaders	2	2
Religious leaders	1	1
District health secretaries	1	0
Total	10	10

### Data collection

Between July and September 2022, we conducted 20 in-depth interviews with key stakeholders from both districts. Prior to these face-to-face interactions, all the participants received an invitation from their respective local government leaders. The interviews with the district health secretaries, HFGC members, their secretaries, and chairpersons took place in their offices, while those with local government leaders and other stakeholders such as religious leaders were held in the offices of the local government leaders. We audio-recorded each interview, which lasted approximately 60 min. An interview guide was used to discuss with participants about their involvement in the committees as a means to enhance social accountability. The interview guide was developed based on the best practices outlined in the 2007 national health policy and the 2013 guidelines, which govern the functioning and operation of the committees as well as the community´s roles within them. The interview covered several topics such as: i) the roles of the HFGCs; ii) the support the HFGC members receive from their represented groups; iii) the selection process for HFGC members; iv) the accountability of the HFGC members to the groups they represent; and v) the linkage of the HFGCs to the local health systems. Throughout the interviews, we utilized probes to motivate discussions, enhance comprehension, and unearth insights from their experiences. Data saturation was determined when no new insights were emerging indicating that additional interviews would not yield new information related to our research questions. We conducted all the interviews in Kiswahili, the native language of all the interviewees.

### Data analysis

We performed a reflexive thematic analysis, following the guidelines outlined by Braun and Clarke [27]. Initially, HAK transcribed the audio recordings of the interviews in Swahili, the original language in which they were collected. Subsequently, HAK and SOM immersed themselves in the data by reading the transcripts multiple times. To ensure transcription accuracy, they cross-checked the transcripts with the original audio and then translated them into English to facilitate analytical discussions within the research group. Next, HAK initiated the coding, aligning the process with the primary objective of the study. The codes were reviewed by the researchers several times until consensus on the codes was reached. Through iterative analysis, several themes were developed, accompanied by brief descriptions that were discussed by the research team. The themes were reviewed and refined several times, with the transcripts revisited as needed until the final four themes were identified and presented in this study.

### Ethical clearance

The research protocol received approval from the National Institute for Medical Research (NIMR) in Tanzania under the reference number NIMR/HQ/R.8a.Vol. IX/3928. Additionally, the President’s Office of Regional Administration and Local Government (PO-RALG), the Regional Administrative Secretary (RAS), and the District Executive Directors from the respective districts granted permission to conduct the study. Participants were approached, and their informed consent to participate in the study was requested. Based on prior experience, verbal consent was preferred, as asking rural respondents to sign formal consent forms can create a barrier between the researcher and the respondent. However, the consent process ensured that each participant understood the purpose of the study and their rights, including the right to withdraw at any time. Additionally, for confidentiality purposes, all data were anonymized, and no personal identifiers were included in the transcripts. All individuals approached for interviews willingly agreed to take part in the research.

## Results

We identified four overarching themes that delineated a series of hurdles hindering stakeholder engagement in the endeavours of HFGCs aimed at fostering social accountability: i) limited awareness of HFGCs among stakeholders and committee members, ii) power dynamics within the committee members and other stakeholders, iii) inadequate political and technical support to the committees; and iv) shortage, but also inefficient use, of financial resources.

### Theme I: limited awareness of HFGCs among stakeholders and committee members

#### Sub-theme: low awareness of HFGCs limits stakeholder participation

The 2013 guideline for the establishment of the CHSBs and HFGCs mandates active community involvement in the activities of the committees, including problem identification, priority setting, seeking funds, and monitoring services at their facilities. However, one of the main reported reasons for the community’s lack of participation in the activities of the committees was their limited awareness of the committees and their roles. As reflected by a district health secretary:

The community does not use these committees because they are not informed about the committees and their functions. (District Health Secretary 1)

Similarly, other participants indicated that the HFGCs were not well known at the mtaa/village level and were confused with other health-related committees formed at that level. As one participant from the local government commented:

I don’t even know about the health facility governing committee, but at the mtaa/village, members of the local government have been divided into various committees such as health, defence, education, and finance. However, I hear some other committees that are normally involved even in providing some vaccines, but I don’t understand how they are formed. (Local Government Leader 4)

#### Sub-theme: insufficient understanding of their responsibilities

Several participants emphasized that a significant challenge influencing stakeholders´ involvement in the committees’ activities was the inadequate comprehension of the responsibilities related to their accountability to the stakeholder groups they represented. Additionally, the lack of recognition by many stakeholders regarding the importance of holding their representatives accountable was identified as a further barrier to establishing effective accountability within the committees. A local government leader expressed clearly these two issues:

The biggest challenges facing these committees first is, the committee members do not know if they are required to be responsible to the community they serve. And second, the community does not seem to understand the importance of these committees being accountable to them. (Local government leader 1)

As an illustration of this situation, the committee members representing facility users who should be accountable to their communities, as outlined by the guidelines, by seeking information before their meetings and providing feedback after their meetings, were not. One participant admitted:

We have never used public meetings to collect opinions from the community, and we do not wait for public meetings to get our agendas for our meetings. Maybe because we did not know that we are required to collect views from the community. (Facility user representative 2)

Participants mentioned several potential explanations contributing to this lack of awareness. Facility managers, for instance, pointed out that the limited educational qualifications of many committee members hindered their ability to fully understand their representative roles, including effectively conveying community concerns. As expressed by one of the secretaries:

The ability to understand things by many members of the committees is still very low due to their low level of education. They were the ones we expected to raise issues from the community, but due to the low level of their understanding, many of the tasks of the committees have fallen on me as the person in charge of the facility. (Secretary 1)

A second main reason mentioned in the interviews was the failure of the district councils to distribute the guidelines to the HFGC members, which some participants identified as a key factor contributing to the members’ limited understanding of their roles. As expressed by several participants:

I suggest that the members of the committees should first be given the guidelines so that they are able to understand their roles because they have not been given the guidelines since they were appointed or selected. (Secretary 4)

On that, there is a challenge, there is a need for us to call our representative in the committee [the local government representative] and instruct him on these functions so that we are able to know what is going on in the committees and work with the committees as well. (Local government leader 2)

The community through their religious leaders observed that the committees´ absence from public meetings impacted awareness of their existence.

The community is not aware of these committees because they do not use public meetings to discuss about what they are doing. (Religious leader 1)

I am a religious leader, I must speak the truth, I do not know the health committees you are asking and I have never seen such committees at public meetings saying anything about their activities. (Religious leader 2)

Despite these challenges, the majority of participants acknowledged the enthusiastic spirit of volunteerism and active participation in the meetings of all members, particularly of women. As noted by the facility manager:

They nearly all attend all the meetings, even in case of emergencies. Once you tell them, even if they are far away, they respond and attend. (Secretary 2)

Currently, women’s participation has improved, and in addition to that, when women get information about meetings, even emergency ones, they are more likely to attend quickly, I think, because of their social responsibilities compared with men. (Secretary 3)

### Theme II: power dynamics within committee members and other stakeholders

#### Sub-theme: flawed selection of HFGC members

The majority of the participants highlighted that the procedures outlined in the 2013 guidelines for selecting HFGC members were not adhered to. For instance, the guidelines specify that community representatives should be selected through a general assembly after posting membership vacancies through the local government office at the mtaa/village level. However, participants observed that local government leaders, especially at the mtaa/village and ward levels, largely controlled and influenced the selection processes. A committee member representing the community remarked that:

I was recommended by my mtaa local government leaders to become a member of the committee. After nomination, the names of all those they appointed as representatives of the facility users were announced and approved during the general assembly. (Facility user representative 1)

Similarly, the selection of HFGC representatives from NGOs/FBOs and private health service providers did not adhere to the guidelines. A member representing drug vendors reflected on her selection process:

As a representative of the drug vendors/pharmacists, I was appointed by the local government leaders. I do not know the procedures used to select members. I was just informed that you have been appointed to be a member of the health committee representing drug vendors/pharmacists. (Drug vendors/pharmacists representatives1)

#### Sub-theme: the secretary assuming the roles of the chairperson

Despite the 2013 guidelines stipulating that the chairpersons of the HFGCs are responsible for convening committee meetings in collaboration with the secretaries, interviewees observed that secretaries were taking on this task and sometimes at short notice. This shift in responsibilities meant that many committee members attended meetings without having sufficient time to gather input from the stakeholder groups they represented. As noted by the chairperson of one of the facilities:

It is the person in charge of the facility who convenes the committee meetings, and regarding the agenda of the meetings, once we arrive, we normally first ask the secretary to tell us what the meeting is about. After getting information about the agenda of the meetings we also get an opportunity to add our own agenda if we have one. (Chairperson 2)

The widespread perception among various stakeholders that secretaries were primarily responsible for all matters related to health facilities beyond their physical premises resulted in many stakeholders inviting the secretaries, rather than the chairpersons, to their meetings for discussion regarding health-related issues. As highlighted by one of the local government leaders:

We have a culture of reviewing reports of all the key institutions in our area to advise each other on various matters regarding our institutions. We invite all the leaders of the key institutions; in the case of the health centre, we usually take the secretary because they are the one who represents the facility including in all other meetings with other institutions. (Local government leader 3)

Another rationale for favouring the secretaries as the spokesperson of the committees was their level of education. As one of the chairpersons commented:

Normally it is the secretary who is given the duty to speak to the community on behalf of the committee, not the chairperson. The secretary acts as the spokesperson of the committee because of their level of education, and education matters, that is why even in many of the meetings of the committees it is the secretary who is more powerful than the chairperson. (Chairperson 4)

### Theme III: inadequate political and technical support to the committees

Despite that the guideline requires the district councils, through their CHMTs, to offer technical advice to the committees and supervise them through the CHSBs, these councils were restricting that support only to the facility managers.

The lack of training and guidelines has caused us to carry out our activities in a business-as-usual manner. (Facility user representative 2)

CHSB and CHMT normally have meetings with us, and we discuss various matters of the centre, but they never go down to the lower level to meet with the committee members to help them in doing their activities, that has never happened. (Secretary 1)

District health secretaries mentioned the shortage of funds as a reason for not carrying out certain responsibilities, like visiting the committees to supervise their activities, by the CHMTs and CHSBs:

The main challenge facing our board is lack of funds to visit and inspect the committees. They do not have enough funds to fuel the cars and visit the committees for supervision. (District health secretary 1)

Furthermore, several participants showed their disappointment for not being formally invited by the local government representatives to the community meetings, limiting the possibility of receiving feedback from the community.

We always attend public meetings organized by the mtaa/village local government leaders, but we only attend the meetings just like any other community members, we are not given invitations or the opportunity to speak to the community as members of the health committee. (Chairperson 1)

No, they do not include us in their meetings, and this denies us the opportunity to hear the community´s concerns about healthcare services. (Drug vendors/pharmacists representative 2)

### Theme IV: shortage, but also inefficient use, of financial resources

Although the committees stakeholders, including NGOs/FBOs, were responsible for assisting the committees in obtaining additional financial resources, this was often not the case. The shortage of funds to support committee activities, particularly in relation to meeting allowances, resulted in the committees relying heavily on the commitment of their members.

One of the challenges we face is the uncertainty of receiving our allowances after meetings. Despite of the uncertainty, we always request the secretary to organize the meetings to ensure community matters are not stalled. Then, when the secretary collects the money from the facility users, he pays us our allowances any day later. (Chairperson 3)

Additionally, it was observed that the failure of many facility managers to adhere to established guidelines contributed to the shortage of funds for financing committee activities, including meetings. This occurred despite the 2020 Comprehensive Council Health Planning (CCHP) guideline, which stipulates that 5% of a facility’s revenue should be allocated to support HFGC activities.

## Discussion

This study explored stakeholders’ participation in the HFGCs to assess their role in promoting social accountability in the local health system of two rural districts of Tanzania. The findings revealed that key stakeholders were neither sensitized nor mobilized to participate in the committees´ activities, making social accountability virtually non-existent. The over-dominance of the facility managers and flawed selection process of HFGC representatives also contributed to weakening the committees as mechanisms for promoting social accountability. Furthermore, the lack of political and technical support from the local government leaders and CHMTs respectively, as well as shortage and inefficient use of financial resources, were key contextual factors that affected the functioning of the committees.

### Committees´ inability to sensitize and mobilize stakeholders

One of the main barriers to the involvement of the stakeholders was an inadequate sensitization and mobilization by their representatives. This was surprising since the guidelines clearly state that the HFGC members should involve their stakeholders in their activities and promote relationships with the stakeholders they represent [16]. The literature suggests that mobilizing a community receiving poor services to come together and demand improvements is a pre-requisite for promoting social accountability [24,28]. The lack of stakeholders´ participation had an additional negative impact on the functioning of the committees, as they did not receive support from these stakeholders, as required by the guidelines [16]. For instance, the limited community participation prevented them from identifying challenges, setting priorities, or monitoring and evaluating services delivered at their facilities [16,17,29]. The reasons why committee members did not mobilize stakeholders were largely due to their limited understanding of their representative role, resulting from inadequate training, supervision, and education, as well as poor dissemination of the guidelines. Evidence from the literature suggests that the capacity and resources of service providers are crucial in promoting social accountability [24,28]. In this situation, capacity building for the health committees should extend beyond simple training sessions [30], to include comprehensive education, provision of guidelines, and ongoing political support [31]. Recognizing the importance of strengthening health committees as legal bodies for overseeing the delivery of health services, the Tanzanian government has emphasized the need for the district councils to provide training, resources, and ensure the recruitment of skilled staff [32]. These councils, through their CHMTs and CHSBs, were tasked with offering technical advice, training, and supervision to the committees but they were not doing it. Although it was not clear why the guidelines were not distributed to the committee members, a lack of funding was identified as a factor preventing CHMTs and CHSBs from visiting committees to provide training and supervision. The lack of training led committee members to rely heavily on secretaries for guidance, resulting in secretaries dominating the committees, a pattern seen in many other LMICs [13,33]. For example, a study conducted in Zambia and Tanzania observed that due to power and information imbalances, service providers were controlling the health committees [34]. Research from Nepal similarly showed that capacity building, training, and clear role definitions for committee members improved community engagement, resource mobilization, and accountability [34]. Additionally, many committee members had low levels of education, as only primary school completion and basic literacy were required for membership [16]. Studies from Tanzania and Kenya have shown that low education levels among committee members affect their performance, including their ability to hold facility staff accountable and engage in effective management [20,35,36]. This issue was also evident in this study, where secretaries ended up dominating the roles of the committees, including responsibilities traditionally held by chairpersons, such as organising meetings. An inadequate understanding of the guidelines also left the committees without sufficient resources for their activities. The 2020 CCHP guidelines for example make clear that 5% of the facility´s revenue should be allocated for committee functions that include meetings, training, and community health promotion activities, but it was not. This observation reflects findings from a study in India that emphasized the importance of distributing guidelines to committee members to enhance their understanding of their roles [37].

Despite the identified challenges, the committees in Tanzania stand out in three key aspects compared to other experiences in SSA. Firstly, unlike in countries such as South Africa and Zambia, where committee members became disillusioned with volunteering because of delays in receiving stipends [12,13,37], our study observed that, despite the uncertainty surrounding the payment of meeting allowances to HFGC members, there was good attendance at the meetings and a positive attitude towards their work. Secondly, while often health committees in many SSA countries tend to be male-dominated [12], women’s representation in the Tanzanian committees was strong, with women exhibiting better attendance and participation than men. One significant contributing factor to this robust participation by women might be the Tanzanian government’s efforts to foster an environment that promotes gender equality in agricultural societies [12,38]. Thirdly, in certain countries, such as South Africa, the lack of formal recognition has negatively affected representation within committees, leading to volunteer fatigue among some members [13]. In contrast, in Tanzania, these committees are formally recognized and act as legal instruments to promote social accountability within the local health system [16].

### The role of contextual dynamics

Several contextual factors affected stakeholder participation in the committee activities to promote social accountability. The appointment of many HFGC members by local government leaders significantly affected their accountability to the stakeholders they represented. Research shows that elected leaders are more responsive to the community, addressing their problems, and meeting their needs, resulting in greater cooperation from the community and in turn, a greater contribution to the general community [39]. Electoral processes that allow stakeholders to choose or remove their representatives make these representatives more accountable due to the fear of being held accountable [40,41]. These findings are consistent with previous research from Tanzania [21,22] and elsewhere [42,43], showing a disconnection between HFGC members and their constituents, due to a lack of community participation in electing their representatives leaving the community unaware of the committees and their roles.

Low political support from local government leaders, particularly at the mtaa/village level, further affected social accountability. These leaders were responsible for ensuring that committees were formed and worked in cooperation with stakeholders [16,17,32]. The failure to connect the committee with stakeholders in community meetings affected stakeholders´ participation in two main ways. It first contributed to making many stakeholders unaware of their representatives and the committees in general. It also denied the committee member opportunities to either collect community views or provide feedback regarding the health service delivery [16]. Additionally, the failure of local leaders to use public meetings to introduce HFGCs led many stakeholders to either not recognize them or confuse them with other multiple health-related committees at the mtaa/village level. Furthermore, it was surprising to find that whenever an issue concerning the health facilities arose, or when general information on their performance was needed, local leaders invited the facility managers, rather than the committee chairpersons, to address the community. Having facility managers address the community undermined accountability mechanisms, as they were unlikely to fully report the challenges the community faced or provide space for open discussion of issues related to the facilities. These findings reflect similar situations observed in Uganda and South Africa, where limited opportunities for community engagement with the committees have hampered their effectiveness in promoting community participation in their activities [44]. In contrast, a study from Uganda showed how health committees achieved significant success by working with local politicians, who provided resources and support that enabled them to engage effectively with the community and raise awareness of health issues [45].

### Methodological considerations

We ensured the trustworthiness of this study in several ways. The use of different approaches enhanced the study’s credibility. First, we designed the interview guides based on the recommended best practices outlined in the guidelines of the roles of stakeholders within HFGCs. Secondly, we collected data from diverse stakeholders within the local health system to capture a range of experiences related to the study topic. Finaly, we employed a member-check approach, where we presented our findings to the participants during a workshop, seeking clarification and validation of our interpretations.

In terms of transferability, the generalization of these findings to other regions of the country warrants careful consideration, given that we conducted the study in only two rural districts. However, it may be reasonable to transfer the findings to other districts in the country, given the similarities in the structure of the local health system across the country. We have provided a detailed description of the contextual characteristics in which we conducted the study, enabling readers to assess the extent to which they can transfer our findings to comparable settings in the country or elsewhere in comparable contexts to Tanzania.

Our grasp of the cultural context, including religion and traditions, and the health system, facilitated our efforts to create an environment where participants could feel comfortable, fostering trust and effective communication. We are aware that establishing rapport and trust necessitates time to develop, potentially restricting the expression of their views and experiences.

Selected quotations extracted from the scripts aimed to illustrate that we could back up our interpretations with the data collected from various diverse participants. Given that we conducted the interviews in Swahili and subsequently translated their content into the English language, we may have lost some meaning from the original narration in the translation process. Nonetheless, the thorough reading by two authors, both native Swahili speakers, enhanced the credibility of the study findings.

## Conclusion

The key stakeholders’ participation in the HFGCs was too weak to promote social accountability in Tanzania’s local health system. The study identified a series of factors contributing to this main finding. First, the lack of awareness about the HFGCs among many stakeholders, as well as the inadequate efforts to sensitize and mobilize them by the HFGCs members, was observed. Second, the committees were unable to handle their representative responsibilities due to insufficient training, supervision, education, and guideline distribution by the district councils. Third, the inadequate election process of HFGC members, interference in committee leadership, insufficient political support from local government leaders, and inefficient use of financial resources further weakened the committees´ capacity to promote social accountability within the health system.

To enhance stakeholder engagement and thus social accountability in the HFGCs, strategies should include increased sensitization and capacity building of both, stakeholders and HFGCs, by the district councils. Furthermore, when approving the facility´s budgets, the councils should ensure that each facility allocates 5% of its budget for committee activities as a condition for approval. The councils should also regularly monitor the performance of the HFGCs and encourage mtaa/village and ward local government leaders to support the committees in engaging with various stakeholders.

## Data Availability

Data are available from Dar es Salaam University (contact via the correspondent author) for researchers who meet the criteria for access to confidential data.

## Abbreviations

CCHP: Comprehensive Council Health Planning
CHMTs: Council Health Management Teams
CHSBs: Council Health Service Boards
CHWs: Community Health Workers
DUCE: Dar es Salaam University College of Education
FBOs: Faith Based Organizations
HFGCs: Health Facility Governing Committees
NGOs: Non-Governmental Organizations
NIMR: National Institute for Medical Research (NIMR)
PHC: Primary Health Care
PO-RALG: President´s Office Regional Administration and Local Government
SSA: Sub Saharan Africa
VCHSC: village council health services committee
